# Predictors of Out-of-Pocket Expenditure on Health Incurred by Elderly Persons Residing in a Rural Area of Faridabad District

**DOI:** 10.7759/cureus.37626

**Published:** 2023-04-15

**Authors:** Vineet Kumar Pathak, Partha Haldar, Shashi Kant, Anand Krishnan, Sanjeev K Gupta

**Affiliations:** 1 Community Medicine, Shree Guru Gobind Singh Tricentenary University (SGT University), Gurugram, IND; 2 Centre for Community Medicine, All India Institute of Medical Sciences, New Delhi, New Delhi, IND

**Keywords:** policy, insurance, rural, health care cost, india, elderly, out of pocket expenditure

## Abstract

Background: A significant portion of India's 1.2 billion population consists of elderly individuals, accounting for approximately 8.6%, who incur substantial out-of-pocket (OOP) healthcare expenses. Any policy for the elderly should encompass financial protection from illness-related expenditures. However, the lack of comprehensive information on OOP expenditure and its determinants precludes such action.

Methods: We conducted a cross-sectional study of 400 elderly persons residing in the rural town of Ballabgarh. The participants were randomly selected using the health demographic surveillance system. We utilized questionnaires and tools to assess the costs associated with outpatient and inpatient services in the previous year, as well as gather information on socio-demographics (individual characteristics), morbidity (motivation for seeking care), and social engagement (health-seeking).

Results: A total of 396 elderly persons participated, with a mean (SD) age of 69.4 (6.7), and 59.4% females. Nearly 96% and 50% of the elderly availed of outpatient and inpatient services, respectively, in the preceding year. The mean (IQR) annual OOP expenditure, as per the consumer price index 2021, was INR 12,543 (IQR, INR 8,288-16,787), with a median of INR 2,860 (IQR, INR 1,458-7,233), explained significantly by sex, morbidity status, social engagement, and mental health.

Conclusion: In low-middle-income countries like India, policymakers may consider pre-payment mechanisms like health insurance for the elderly, using such prediction scores.

## Introduction

India is currently witnessing a decline in fertility rates and an increase in life expectancy, leading to a rise in the absolute number and proportion of elderly people aged 60 years and above. In 2011, India's elderly population was 103.8 million, which is the second-largest in the world after China. It is projected to increase to 140 million in 2021 and 315 million by 2050 [1]. However, the gross domestic product (GDP) allocated to the health sector in India is only 3.9%, in comparison to high-income countries that spend 11.9% of their GDP on healthcare [2]. India also has a high proportion of private health expenditure, with 72% of total health expenditure being contributed by private sources, of which 69% is out-of-pocket (OOP) expenditure [3]. This lack of comprehensive insurance schemes, combined with high OOP payments in public health sectors and the increased preference for healthcare seeking in private health sectors, creates inequities in healthcare access in India [4].

Regarding elderly individuals, most of the diseases affecting them are chronic and require longer treatment duration. They are also more prone to developing multiple comorbidities, resulting in higher healthcare expenditures than other age groups. Moreover, only a small proportion (14.1%) of the Indian population has any form of health insurance [5], leaving elderly individuals with the burden of OOP expenses when they fall ill. Research indicates that catastrophic illnesses can push Indian families into indebtedness [6]. The affordability and accessibility of health services place this age group at higher risk of poor health outcomes. Inequities in access to health services, health payments, and healthcare distribution are significant public health issues in Asia [7].

However, in India, there is very limited data available on the amount of OOP expenses that are being incurred by elderly individuals. This lack of comprehensive information precludes policymakers from reforming existing social security schemes and developing models to protect elderly individuals from financial hardship. Identifying expenses incurred through healthcare insurance to provide targeted preventative health interventions and financial protective interventions through prepayment schemes is crucial. Moreover, healthcare expenditure among the elderly is influenced by several factors, and it is essential to develop a comprehensive framework that includes the measurement of social isolation, morbidity, and mental health status. The present study was conducted among the 28 villages of the Health Demographic Surveillance System (HDSS), a model of rural healthcare practice in India with the objective of estimating the annual OOP expenditure on health incurred by elderly persons residing in the rural part of Ballabgarh town.

## Materials and methods

Study site

A community-based cross-sectional study was conducted from May 2016 to June 2016 across 28 villages comprising the rural area of a medical teaching institute in Northern India [8]. A list of all elderly persons aged 60 years and above was obtained from a computer-based Health Management Information System (HMIS) in the area, which maintains demographic details of the population. This was used as the sampling frame. Study participants were selected by simple random sampling from the sampling frame. The selected elderly persons were aged 60 years and above, residing in the study area for at least six months, and able to comprehend the questions, i.e., understand when asked in the local language and not too sick to answer the questions at the time of the interview. Ethical approval was received from the Ethics Committee of All India Institute of Medical Sciences (AIIMS), New Delhi (IECPG/79/30.12.2015). Informed written consent was obtained from all participants following the administration of a participant information sheet. The study was conducted according to the principles laid out in the Declaration of Helsinki.

Sample size calculation

The sample size was calculated using the formula to estimate the annual mean expenditure. An earlier study conducted by Ray TK et al. [9] in the same study area reported a mean (SD) annual expenditure among the elderly of INR 117.8 (71.9). Considering a level of significance of 5%, with an absolute precision of INR 8, the minimum required sample size was 323. Assuming a non-response rate of 20%, the required sample size was 387, which was rounded off to 400 study participants.

Study tools

**Figure 1:**
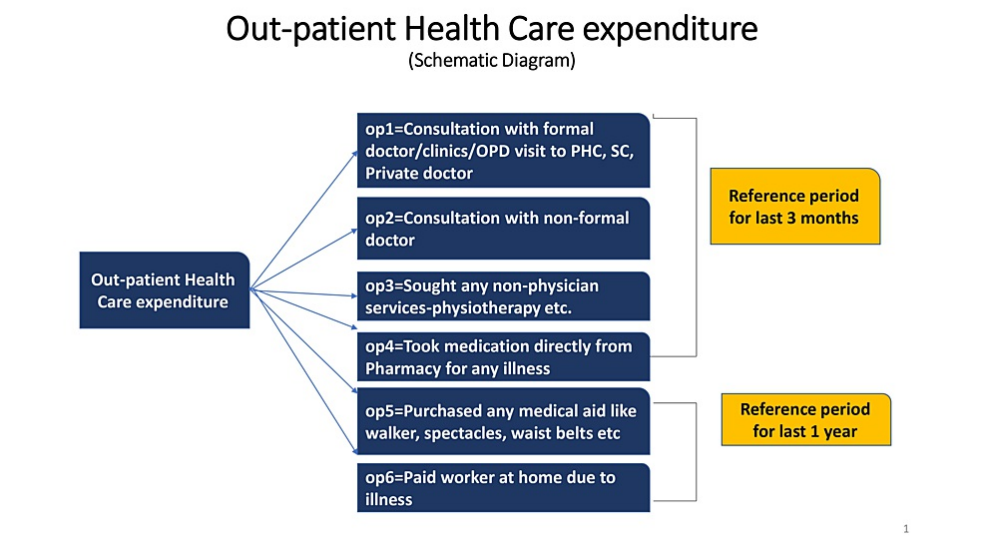
Schematic flow diagram showing the distribution of outpatient health care expenditure into six subheadings, their reference period, and calculation of annual outpatient expenditure op: outpatient, OPD: outpatient department, PHC: primary health centre, SC: subcentre

**Figure 2:**
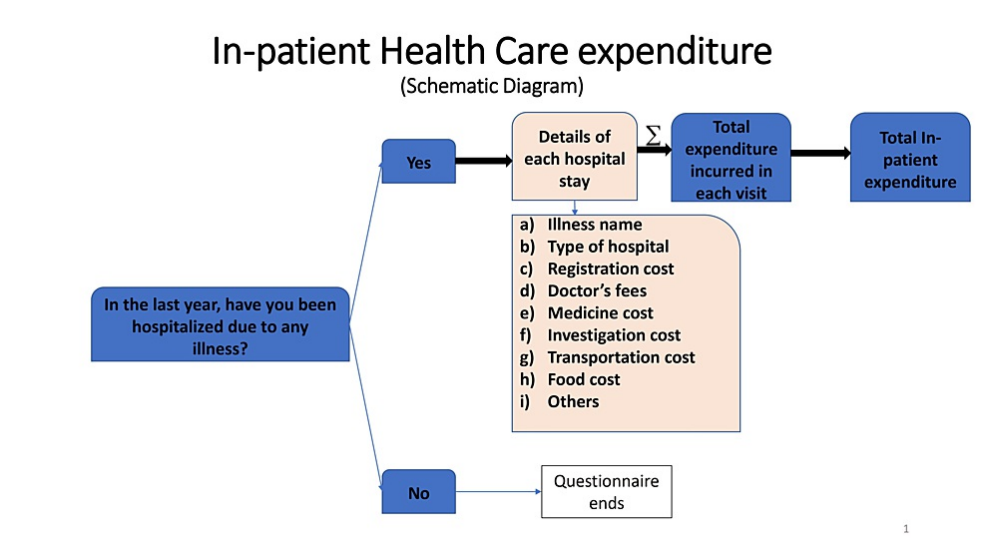
Schematic flow diagram showing the distribution of inpatient health care expenditure, details asked during each hospital stay, and calculation of annual inpatient expenditure

We developed a semi-structured questionnaire to collect information on socio-demographic characteristics, healthcare utilization patterns, and healthcare expenditure on available outpatient and inpatient health services. OOP expenditure was defined as direct payments made by participants to healthcare providers at the time of service use. This excludes any prepayment for health services, for example, in the form of taxes or specific insurance premiums or contributions and, where possible, net of any reimbursements to the individual who made the payments [10]. All expenditures were calculated in Indian National Rupee (INR) at Consumer Price Index (CPI) 2016 and then converted at CPI 2021 price levels for this manuscript writing. Outpatient care referred to ambulatory care received in the previous three months, excluding situations where the patient was allotted a bed/trolley for treatment (irrespective of the duration of time). Outpatient expenditure was further subdivided into six domains as follows: expenditure incurred for consultation with a formal doctor, consultation with a non-formal doctor, availing non-physician services such as physiotherapy, occupational therapy, accessing pharmaceutical items, and medical aid expenses and paid worker at home due to any illness (Figure 1). Inpatient care referred to a stay in a hospital/clinic where the patient was allotted a bed/trolley for treatment irrespective of the duration of time spent in that facility, in the last year from the date of data collection. Inpatient expenditure was also asked in detail as a description of each stay in the last year (Figure 2).

The different time frames for outpatient and inpatient care were used because an inpatient event is a significant period in one's life, and recall is possible even when asked about the past year, which might not be possible for quick outpatient visits for minor ailments in older persons. A person is likely to spend on healthcare services when there is a need in terms of morbidity. In addition to need factors, studies [11] have shown that there are other social factors that can affect healthcare access, such as social engagement. We used the Lubben Social Network Scale (LSNS) [12] to capture the social engagement of the participants. The Lubben Social Network Scale-6 (LSNS-6) is a six-item, self-reported scale to assess social isolation. Scores range from 0 to 30, i.e., each question has a response from 0 to 5. The LSNS is a continuous scale ranging from a minimum score of 0 to a maximum of 30. The lower the score signifies the more risk of social isolation. The Cumulative Illness Rating Scale for Geriatrics (CIRS-G) [13] captures multimorbidity and the severity of morbidity. The CIRS-G is a modified version of the Cumulative Illness Rating Scale, which is a well-established measure of multimorbidity among the elderly. On the CIRS G, 13 categories referring to clinically relevant physiological systems and one category referring to psychiatric illness are rated on a five-point severity scale. The SF-12 (Short form survey) [14] assesses the limitation in role functioning as a result of physical and emotional health.

Data analysis was done using Stata software version 12 (Stata Corporation LLC, College Station, USA). It was log-transformed to normalize the dependent variable, i.e., annual OOP expenditure. After applying regression, the results were exponentiated. The chi-square test was used for categorical variables. Bivariate Linear regression was done for all independent variables, and the variables with p-value< 0.05 were put in the multiple linear regression model. Highly correlated independent variables were removed from the multiple linear regression model to deal with multicollinearity. Results were expressed in mean, median, p-value, 95% CI, and the standard error (b_SE) was calculated using the bootstrapping function. The bootstrap is used in statistics as a resampling method to approximate standard errors, confidence intervals, and p-values. A p-value <0.05 was considered statistically significant.

## Results

A total of 161 men (40.6%) and 235 women (59.4%) completed the study. Table 1 describes the socio-demographic and health-related characteristics of the participants. The mean age of both men and women was 69.4 ± 6.7 years. The majority of participants were illiterate (65.9%) and 64.7% were married and living with their spouses, while 34% were widowed. Agriculture was the most common past occupation (47.8%), and only 12.1% of the participants belonged to a nuclear family. Nearly one-third of the participants had a family size of 5-6 members. Most participants (86.4%) were receiving an old-age pension scheme, but only 7.6% had government or private health insurance. About 38.4% reported that major family decisions, such as marriage, sale, or purchase of land, were taken jointly by family members.

**Table 1:** Socio-demographic and health-related characteristics of study participants ^a^ Difference of proportions: chi-square test was used, where value <5 Fisher’s exact test was applied; ^b^ Numbers rounded off to nearest whole number. LSNS: Lubben Social Network Scale; CIRS-G: Cumulative Illness Rating Scale-Geriatrics; IQR: Inter Quartile Range; SF-12: Short Form-12

Parameter	Total n=396
Age	
Mean (SD)	69.4 (6.7)
Education: n (%)	
Illiterate	261 (65.9)
Less than high school	80 (20.2)
High school and above	55 (13.9)
Marital status: n (%)	
Married and staying with spouse	256 (64.7)
Widow/widower	135 (34.0)
Others (separated, never married)	5 (1.3)
Occupation: n (%)	
Farmer	188 (47.8)
Government job	27 (6.8)
Private job	30 (7.6)
Labourer	48 (12.2)
Others (Business, Homemaker)	103 (25.6)
Family type: n (%)	
Nuclear	48 (12.1)
Extended	348 (87.9)
Monthly household income: INR (SD)^a^	8,701 (4,556)
Per capita income: INR (SD)^b^	1,398 (885)
Availing social security scheme: n (%)	
Yes	342 (86.4)
No	54 (13.6)
Availing any health insurance: n (%)	
Yes (Government/Private)	30 (7.6)
No	366 (92.4)
Decision maker in the family: n (%)	
Self	79 (20)
Mainly children	126 (32)
Mainly spouse	38 (9.6)
Jointly	153 (38.4)
LSNS Score:	
Mean (SD)	13.7 (5.2)
Median (IQR)	14 (11-17)
CIRS-G Score:	
Mean (SD)	7.3 (3.7)
Median (IQR)	7 (5-9)
SF-12 MCS Score:	
Mean (SD)	26 (14.9)
Median (IQR)	21.8 (15.8-36.3)

The mean LSNS score was 13.7 (SD 5.2), with a median score of 14 (IQR 11-17). The mean CIRS-G score was 7.3 (SD 3.7), with a median score of 7 (IQR 5-9). The severity index (total score divided by the number of categories endorsed) for CIRS-G was 1.51 (SD 0.35), with a median of 1.5 (IQR 1.3-1.7). The mean SF-12 MCS score was 26 (SD 14.9), with a median score of 21.8 (IQR 15.8-36.3).

The distribution of study participants on the expenditure of outpatient services is described in Table 2. Out of the 396 participants, 381 (96.2%) availed outpatient services, while 201 (50.7%) availed inpatient healthcare services during the reference period of this study. The total expenditure incurred by the participants for availing the outpatient services was further distributed as a visit to formal doctors (82.3%), over-the-counter purchase of medicine (55.3%), medical aid use (26%), and visit to a non-formal doctor (13%). The mean (95% CI) annual outpatient OPP expenditure was INR 4,616 (3,455-5,876) with a median of INR 1,878 (IQR, INR 775-4,137).

**Table 2:** Distribution of study participants on the expenditure of outpatient services ^a^ Bootstrapping standard error (SE) based on 500 replications; ^#^ More than one service availing by single participants; values are rounded off to the nearest whole number. INR: Indian National Rupee; CI: Confidence Interval; IQR: Inter Quartile Range

S. No.	Outpatient Services	No. of Participants N, %	Mean (in INR)	b_SE^a^ (in INR)	95% CI (in INR)	Median (in INR)	IQR (in INR)
1	Visit to formal doctor	326 (82.3%)	4,341	711	2,934-5,786	1,265	651-3,675
2	Visit to non-formal doctor	52 (13.1%)	1,175	299	598-1,766	651	217-1,092
3	Non-physician services	8 (2%)	1,482	321	868-2,125	1,302	1,182-1,639
4	Medication direct from pharmacy	218 (55%)	1,025	134	763-1,294	523	209-965
5	Medical aid expenses	103 (26%)	411	74	269-554	217	164-329
Annual out-of-pocket expenditure outpatient		707^#^	4,431	613	3,308-5,621	1,796	741-3,944

The mean (95% CI) expenditure for those who sought care from a formal doctor was INR 4,531 (3,059-6,040). This was further subdivided as medicine cost (63.0%), doctors’ fees (20.3%), investigation cost (10.4%) i.e. cost incurred on undertaking diagnostic test advised by a doctor, registration cost at the hospital/clinic (4.1%), transportation cost (2.0%), and others (2.1%). The mean (95% CI) expenditure for those who had visited a non-formal doctor was INR 1,226 (625-1,845). The salient reasons for visiting a non-formal doctor were for long-term illnesses such as knee pain, recurrent cough, diabetes, generalized weaknesses, etc. The mean (95% CI) expenditure for those who utilized any non-physician services was INR 1,549 (907-2,223); these were mainly for physiotherapy for knee pain, chronic backache, etc. The mean (95% CI) expenditure for the over-the-counter purchase of medicines was INR 1,073 (798-1,359); medicines were purchased for long-term illnesses such as diabetes, hypertension, medication for eye problems, analgesics for chronic knee pain, cough, nutritional supplements such as vitamins, etc. The mean (95% CI) expenditure incurred on the purchase of medical aid was INR 430 (281-578); the walker, waist belts, portable nebulizer and inhalers, spectacles, dentures, etc.

The distribution of study participants on expenditure for inpatient services is described in Table 3. The mean annual OOP expenditure (95% CI) for inpatient services was INR 7,343 (3,967-10,711) with a median of INR 247 (IQR, INR 0-1,616). Almost 92% of the participants availed of private hospitals/clinics services during their inpatient service utilization. This high cost may be due to the fact that almost half (50%) of the participants had at least one inpatient event in the past year, as per our inpatient operational definition, and the subsequent inpatient admission rate was lower in the same year. The cost of inpatient admission was further subdivided into medicines (54.8%), doctor’s fees (21.1%), investigations within the facility (9.8%), registration (1.2%), food (1.0%), and others (10.7%). The category of 'others' included the purchase of medical aids and devices, such as stents for angioplasty, lenses for cataract surgery, staplers for haemorrhoidectomy, bone nails, K wires, etc.

**Table 3:** Distribution of study participants on the expenditure of inpatient services ^a^ Bootstrapping standard error (SE) based on 500 replications; ^#^ More than one service availing by single participants; values are rounded off to the nearest whole number. INR: Indian National Rupee; SD: Standard Deviation; IQR: Inter Quartile Range

S. No.	Inpatient Services	No. of Participants N, %	Mean (in INR)	b_SE^a^ (in INR)	95% CI (in INR)	Median (in INR)	IQR (in INR)
1	1^st^ Hospital admission	201 (50.7%)	13,361	3,286	6,924- 19,791	1,369	763-5,479
2	2^nd^ Hospital admission	24 (6.0%)	7,822	2,672	2,575-13,062	1,235	681-11,026
3	3^rd^ Hospital admission	4 (1.0%)	8,870	6,587	4,042-21,782	2,327	621-17,111
Annual out-of-pocket expenditure inpatient		229^#^	7,343	1,811	3,967-10,711	247	0-1,616

A bivariate linear regression with socio-demographic variables and LSNS, CIRS-G, and SF12 scale scores are described in Table 4 and Table 5. A simple linear regression was calculated to predict the annual OOP expenditure based on socio-demographic variables, and the scores of LSNS, CIRS-G, and SF12. The variable, decision maker in the family, and the scores of LSNS, CIRS-G, and SF12 were found to be significantly associated with the annual OOP expenditure.

**Table 4:** Bivariate linear regression of annual out-of-pocket expenditure with socio-demographic variables

Independent Variable	No. of Participant (N=396)	ß Coefficient	95% CI	P-value	
Age Group					
60-64 years	108	Ref			
65-74 years	189	1.1	0.7-1.5		0.64
>=75 years	99	1.2	0.8-1.8		0.27
Sex					
Men	161	Ref			
Women	235	0.9	0.6-1.2		0.67
Marital status					
Married and staying with spouse	256	Ref			
Widow/widower	135	0.7	0.09-5.8		0.77
Married and not staying with spouse	3	1.0	0.07-14.3		0.99
Never married	2	0.7	0.09-5.7		0.76
Educational status					
Illiterate	261	Ref			
Upto Primary school pass	35	1.5	0.9-2.6		0.11
Less than high school certificate	45	0.9	0.5-1.4		0.75
High school certificate	36	0.9	0.5-1.5		0.70
Secondary certificate and above	19	1.6	0.8-3.1		0.18
Family size					
1-2	30	Ref			
3-4	33	0.8	0.4-1.7		0.59
5-6	119	1.1	0.6-2.0		0.68
7-8	111	1.2	0.6-2.1		0.56
>=9	103	1.5	0.8-2.6		0.21
Occupation					
Farmer/cultivator	188	Ref			
Government job	27	1.07	0.5-1.9		0.81
Private job	30	1.2	0.7-2.2		0.43
Business	22	1.3	0.6-2.5		0.42
Labourer	48	1.5	0.9-2.4		0.08
Homemaker	81	1.05	0.7-1.5		0.78
Family type					
Nuclear	48	Ref			
Extended	348	1.2	0.8-1.9		0.29
Monthly household income (in INR)					
<6000	135	Ref			
6001-8000	102	1.06	0.7-1.5		0.7
8001-10000	71	0.99	0.6-1.5		0.9
≥10001	88	1.20	0.8-1.7		0.3
Any kind of social security scheme					
No	366	Ref			
Yes	30	0.99	0.6-1.5		0.9
Health insurance					
No	366	Ref			
Yes	30	1.4	0.8-2.5		0.19
Decision maker in the family					
Self	79	Self			
Mainly Children	126	1.3	0.9-2.1		0.1
Mainly spouse	38	2.0	1.1-3.5		0.01
Jointly	152	1.3	0.8-1.9		0.2
Others	1	3.1	0.1-57.2		0.4

**Table 5:** Bivariate linear regression of annual out-of-pocket expenditure with LSNS, CIRS-G, and SF12 Scale LSNS: Lubeen Social Network Scale; CIRS-G: Cumulative Illness Rating Scale-Geriatric; SF12: Short Form-12

	ß Coefficient	95% CI	P-value	Prob>F value	R-squared
Lubben Social Network Score	1.04	1.02-1.06	0.036	0.03	0.01
Cumulative Illness Rating Score	1.13	1.09-1.18	<0.001	<0.001	0.09
Mental Component System Score of SF12 Scale	1.03	1.02-1.04	0.001	<0.001	0.08

Multiple linear regression with variables that are significant in the bivariate analysis is shown in Table 6. Multiple linear regression analysis was used to test the predictors of the annual OOP expenditure, out of the variables that were significant in bivariate analysis, along with the inclusion of age and sex, despite the latter two being non-significant in univariate analysis. The results of the multiple linear regression indicated that the five predictors, viz, sex, the decision maker in the family, LSNS, CIRS-G, and mental health component of the SF12 explained 16% of the variance (R2=0.16, F (5/382) = 12.91, p<0.0001). It was found that LSNS (b=1.04, p=0.004), CIRS-G (b=1.13, p<0.001), and the mental health component of the SF12 (b=1.03, p=0.001) independently predicted the annual OOP expenditure in this study population.

**Table 6:** Multiple linear regression of annual out-of-pocket expenditure with variables that are significant in bivariate analysis (p<0.05), age group, sex, decision-maker

Independent Variables	ß Coefficient	95% CI	P-value	Prob>F	R-squared	
Age group				<0.001		16%
60-64 years	Ref					
65-74 years	1.01	0.7-1.4	0.91			
>=75 years	0.97	0.6-1.4	0.91			
Sex						
Male	Ref					
Female	0.69	0.5-0.9	0.02			
Decision maker in the family						
Self	Ref					
Mainly children	1.21	0.8-1.8	0.36			
Mainly spouse	1.8	1.0-3.4	0.04			
Jointly	1.1	0.8-1.7	0.37			
Others	3.2	0.2-50.1	0.39			
Lubben Social Network Score	1.04	1.01-1.06	0.003			
Cumulative Illness Rating Score	1.10	1.05-1.14	0.003			
Mental Component System of SF12 Score	1.02	1.06-1.02	<0.001			

## Discussion

Currently, there is a lack of studies available on the estimation of OOP expenditure among elderly individuals living in rural areas of India. Therefore, our study serves a critical purpose by providing insight into the annual OOP expenditure on healthcare incurred by elderly individuals, as well as breaking down this cost into outpatient and inpatient categories.

In our study, the mean annual OOP expenditure was INR 12,543, or INR 1,045 per month. When comparing our findings to studies conducted outside of India, we found that Heider D et al.'s study in Germany reported a mean total expenditure on health of 889 € (INR 87,131) for a three-month recall period for the age group 57-84 years. This amount appears quite high on an absolute scale, especially when compared to our study. One possible explanation for this difference is that nearly 92% of participants in Germany had health insurance, while only 7% of our study had any form of health insurance. Additionally, Germany has a higher standard of living and spends nearly 10 times more of its GDP on health than our country.

Another study conducted in Mexico in 2006 [15], which was a secondary data analysis of the Mexican National Health and Nutrition Survey (ENSANUT), reported household expenditure during hospitalization for elderly individuals living with them as $308.9 (INR 23,121). This difference in expenditure may be due to the fact that they reported household expenditure rather than individual expenditure, as we did in our study. Furthermore, the coverage of health insurance in Mexico is nearly 89%, and they spend 6.4% of their GDP on health.

A study conducted in California, USA [16] analyzed secondary data from 7,836 participants aged ≥65 years from the Health Retirement Study (HRS) and reported a mean OOP expenditure in the HRS over a two-year period of USD 2,022 (INR 1,51,351) with a median of USD 920 (INR 68,864). When compared to Indian studies, such as a community-based study conducted among 314 individuals [17], the mean (SD) OOP expenditure among elderly individuals was reported as USD 6.1 (4.5) per month (INR 456,336). The differences in reported expenditure could be attributed to different study settings and parameters used to assess expenditure. The study conducted by Shukla M et al. included only expenditure for consulting allopathic and AYUSH doctors and calculated monthly expenditure, whereas we assessed outpatient expenditure annually and comprehensively under five domains.

In our study, the private sector accounted for most expenditures incurred in outpatient (74.7%) and inpatient services (97.7%). A study conducted among the elderly [18] reported that 81.2% preferred allopathic medicines, followed by Ayurvedic medicines (11.3%). Additionally, another study [19] reported that only 30% of elderly persons preferred allopathic medications for their illnesses; the majority (70%) preferred the alternative system of medicine (AYUSH, especially Ayurveda and Homeopathy). The variation in choosing the type of health system could be due to the availability and accessibility of healthcare systems in a particular area and may vary for different geographical areas.

During the study period, 1279 episodes of illness were recorded, including outpatient and inpatient services from both the public and private sectors. The mean expenditure incurred on utilizing outpatient services per episode was USD 22.5 (INR 1,684), which was similar to the study conducted by Raykarmakar P et al. [20] where they reported a mean outpatient expenditure per episode of USD 21.4 (INR 1,601). In our study, the mean annual outpatient expenditure was USD 59.7 (INR 4,468), which translates into USD 4.9 (INR 366) per month. Outpatient expenditure was maximum for medicines (63%), doctor’s fees (20%), investigation (10%), and transportation (3%). Another study [21] reported expenditure on medicines (55%), doctor’s fees (10%), and transportation (10%). A study conducted [22] also reported that most of the OOP expenditure was for doctor’s fees (31.6%) and medicine costs (31.7%). Also, Longitudinal Ageing Study in India (LASI) 2020 [23] report has reported that the mean OOP expenditure of the elderly on outpatient care in one month time period is USD 14 (INR 1,149) which is nearly equal to our mean annual OOP expenditure.

Our study reports the mean annual inpatient expenditure as USD 98.1 (INR 7,343). A study conducted by Archana et al. reported an expenditure of USD 19.6 (INR 1,467) as the hospitalization cost in the last three months. This difference could be because they had calculated the expenditure only for three months and also because the study group was at the household level and not exclusively elderly.

## Conclusions

This community-based study utilized the CIRS-G scale, a tool better than self-reported morbidity, to collect morbidity data. Additionally, an MBBS doctor applied the CIRS-G scale, improving the collected data quality since data collection requires medical background knowledge. We used the LSNS scale to examine the association of social engagement of elderly persons, which indirectly influences their health expenditure. Finally, we developed a comprehensive questionnaire to capture as many domains as possible related to health expenditure. We acknowledge the limitations of the study, including the non-availability of medical bills, prescriptions, and other medical records for some participants, which could lead to inaccuracies in capturing OOP expenditures. Very old elderly persons may also have recall bias even for the past three months and may be unable to provide expenditure details. Moreover, we identified the exponentiation of outpatient expenditure annually by multiplying it by four as a limitation of the present study. As a cross-sectional study, we could not estimate the impact of seasonal variation on elderly health and related expenditure.

Our study provides evidence of the cost burden to inform existing policies related to the old age pension and personal private health insurance coverage, especially in the elderly age group. Our findings emphasize the need for a systematic and sustainable plan to provide healthcare services for the elderly population. Based on our study's findings, we recommend that policies for the healthcare of elderly persons in India should consider financial protection like free OPDs/IPDs and dedicated elderly clinics since most users spend OOP, and the use of private providers is also substantial. The policy should consider a pre-payment mechanism such as insurance as one of the components for financial protection.
